# Supplementary material for: Genomic Epidemiology of SARS-CoV-2 in Seychelles, 2020–2021
Source: Viruses. 2022 Jun 16;14(6):1318. doi: 10.3390/v14061318 (PMC9231335; doi:10.3390/v14061318)
Supplement: Supplementary file 1 [file viruses-14-01318-s001.zip › SupplementaryFigureS2.pdf]

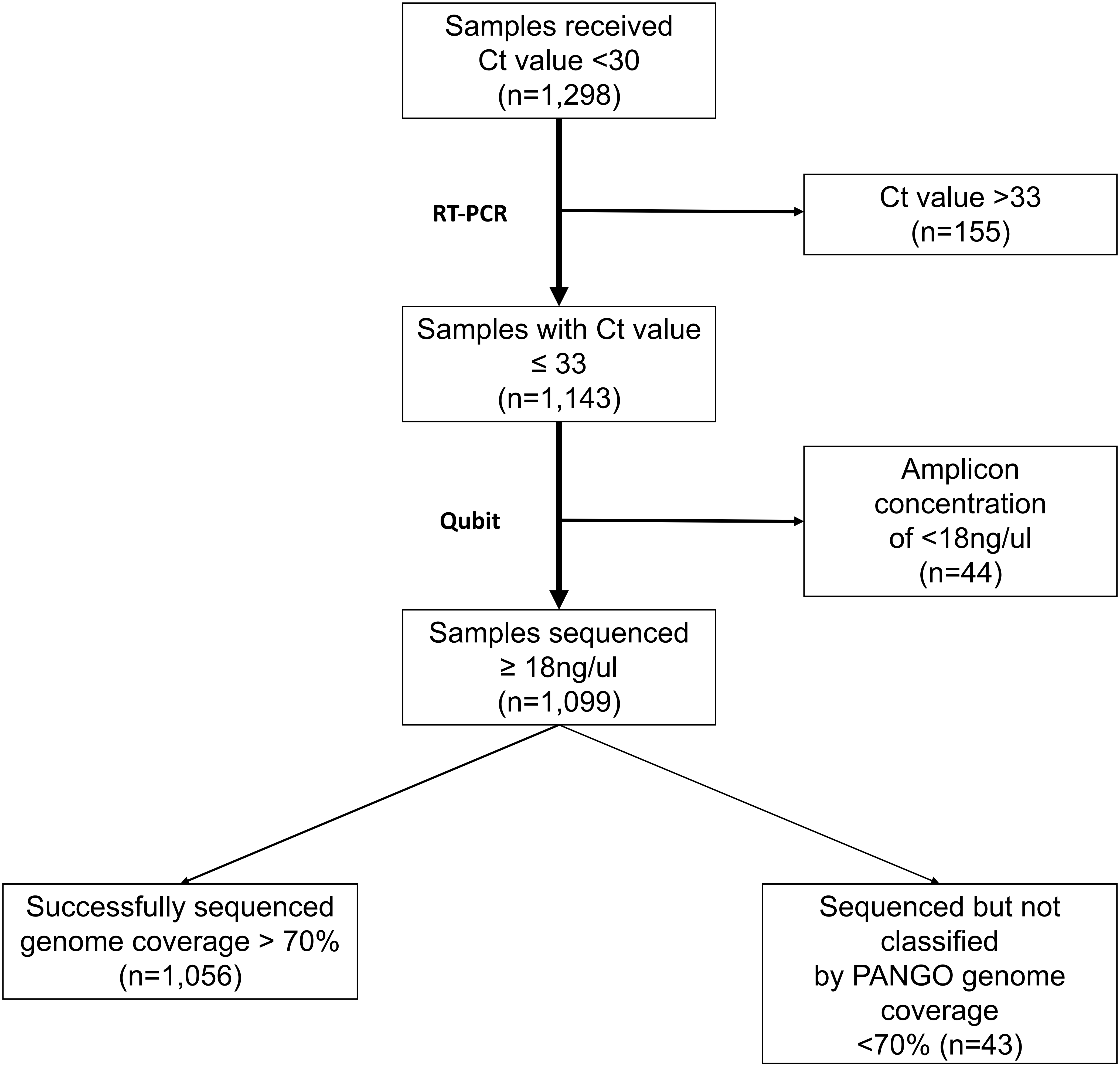

Samples received
Ct value <30
(n=1,298)
RT-PCR
Ct value >33
(n=155)
Samples with Ct value
≤ 33
(n=1,143)
Qubit
Amplicon
concentration
of <18ng/ul
(n=44)
Samples sequenced
≥ 18ng/ul
(n=1,099)
Successfully sequenced
genome coverage > 70%
(n=1,056)
Sequenced but not
classified
by PANGO genome
coverage
<70% (n=43)
