# Supplementary material for: Genomic Epidemiology of SARS-CoV-2 in Seychelles, 2020–2021
Source: Viruses. 2022 Jun 16;14(6):1318. doi: 10.3390/v14061318 (PMC9231335; doi:10.3390/v14061318)
Supplement: Supplementary file 1 [file viruses-14-01318-s001.zip › SupplementaryTableS1.pdf]

Supplementary Table S1. Number of viral imports and exports using different sub-samples sets.

	No of sequences	Number of viral imports	Number of viral exports
Sub-sample 1	5,179	78	32
Sub-sample 2	5,211	74	34
Sub-sample 3	5,218	75	35
