# Supplementary figures and images for: Genomic Epidemiology of SARS-CoV-2 in Seychelles, 2020–2021
Source: Viruses. 2022 Jun 16;14(6):1318. doi: 10.3390/v14061318 (PMC9231335; doi:10.3390/v14061318)

**A**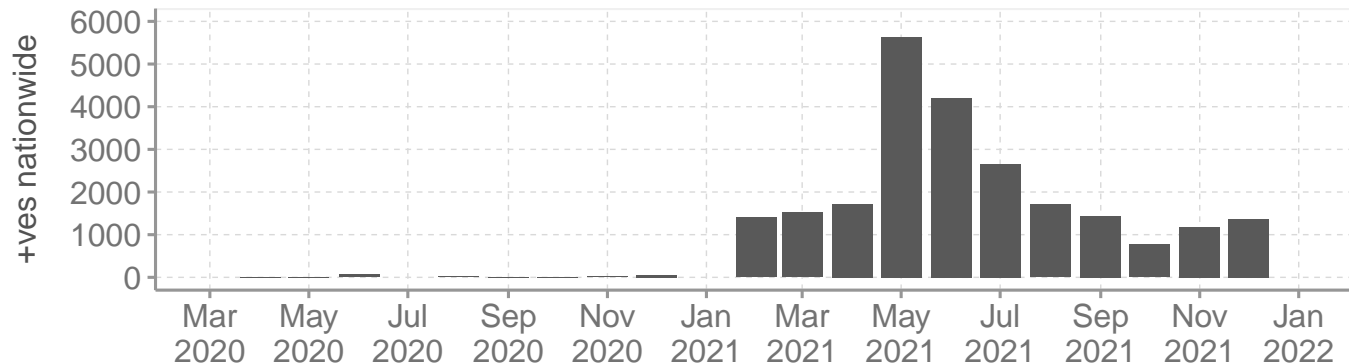**B**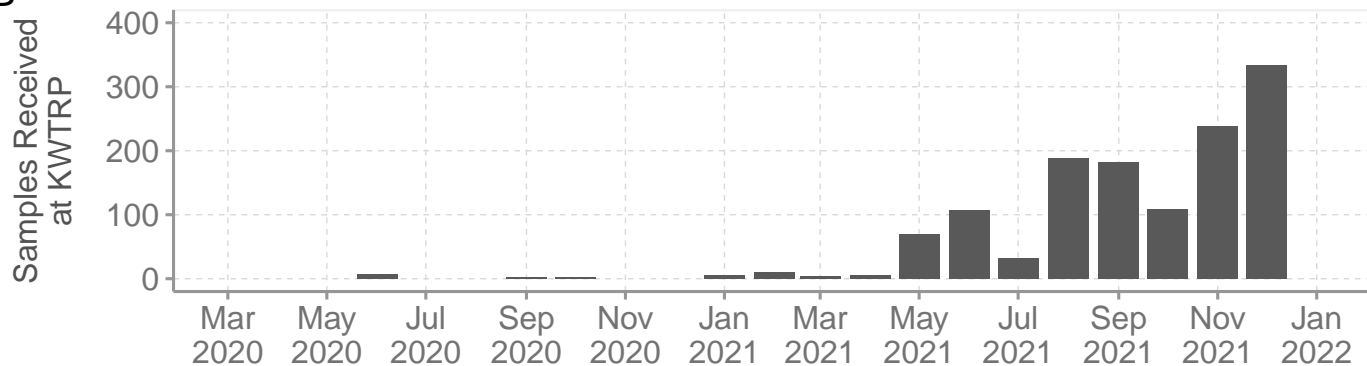

Supplement: Supplementary file 1 [file viruses-14-01318-s001.zip › SupplementaryFigureS1.pdf]

A

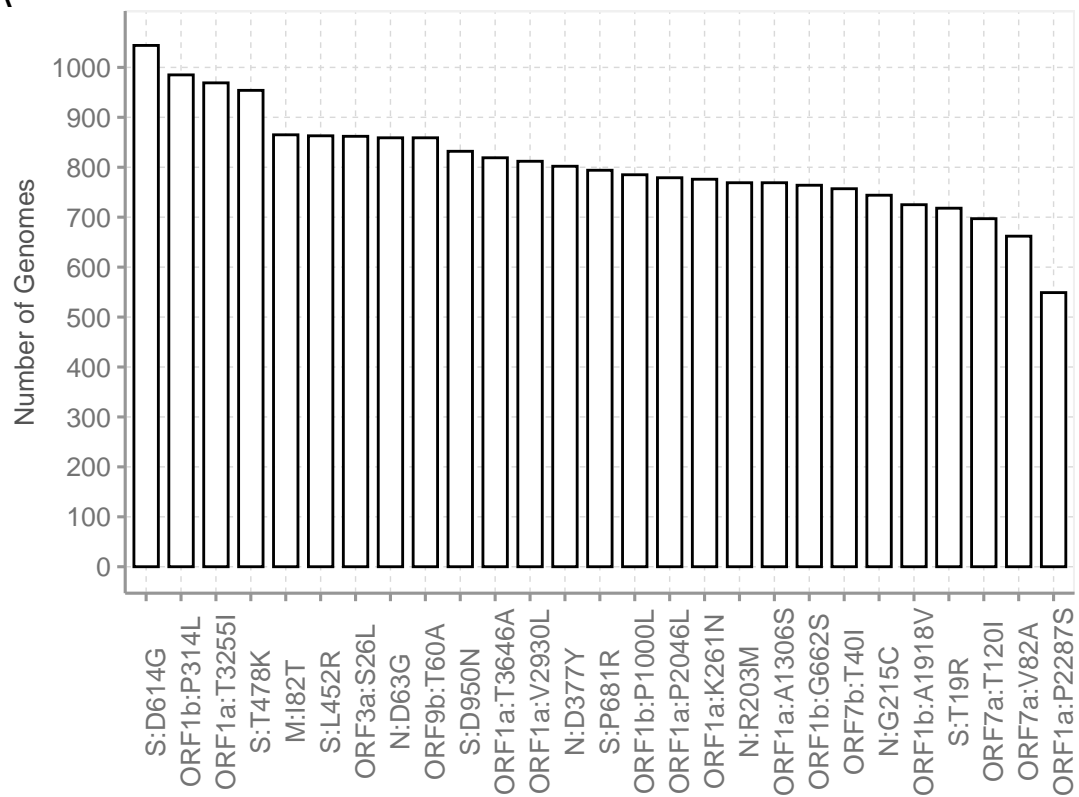

B

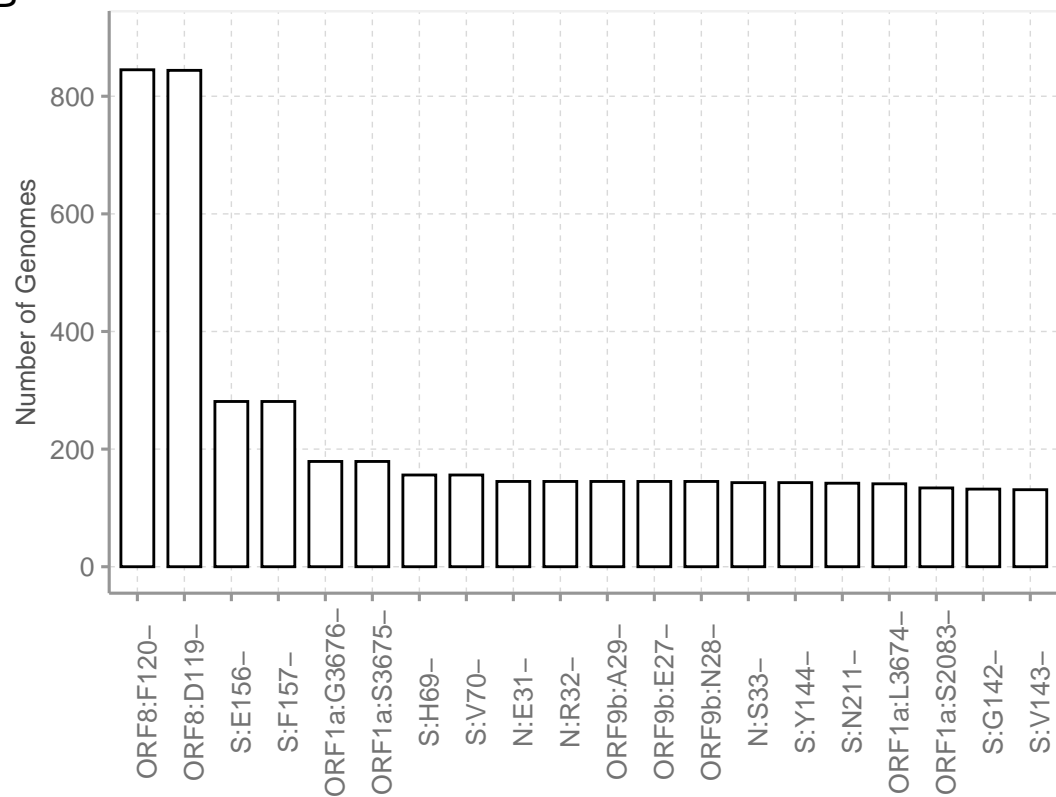

C

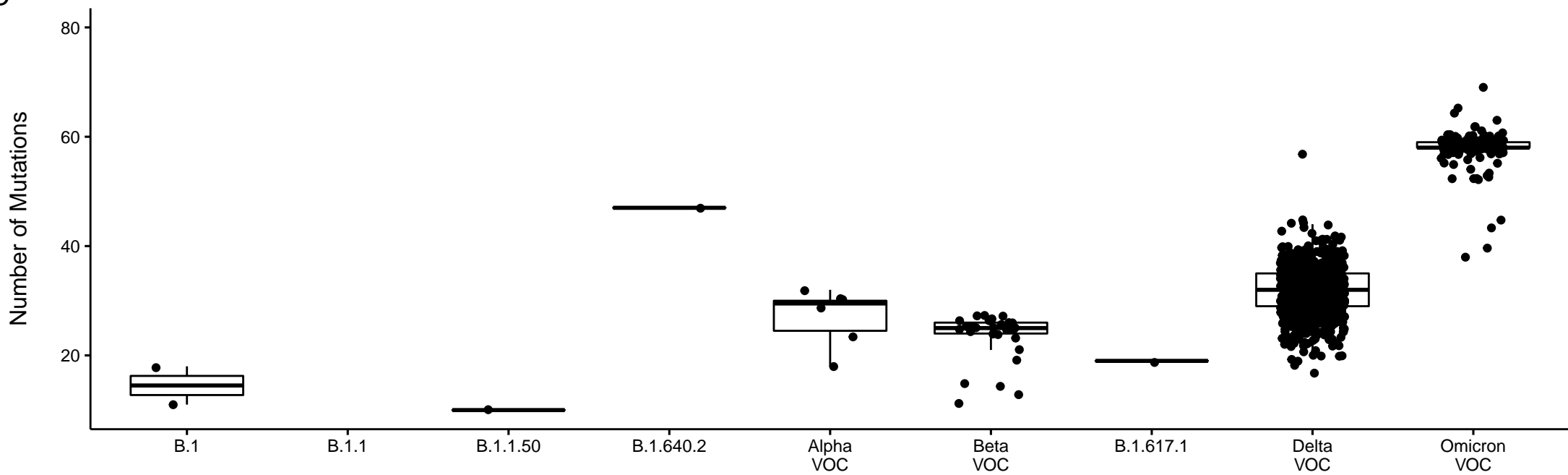

Supplement: Supplementary file 1 [file viruses-14-01318-s001.zip › SupplementaryFigureS3.pdf]
